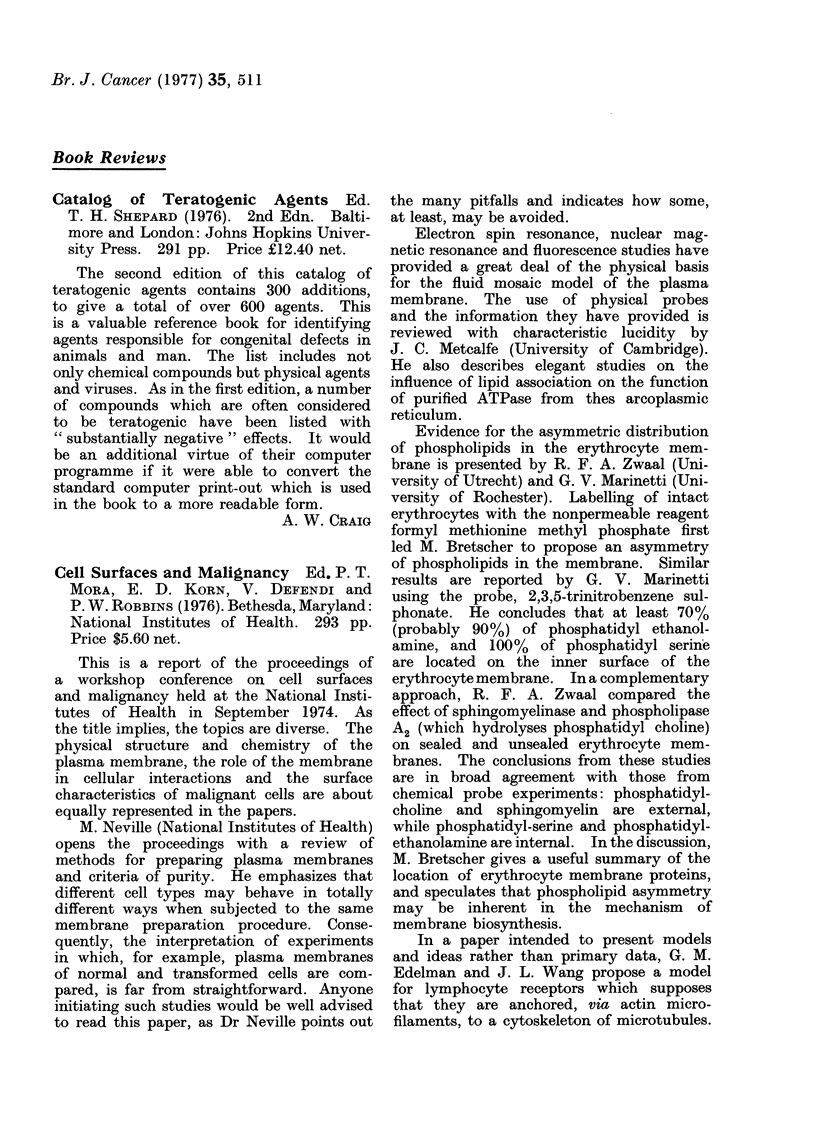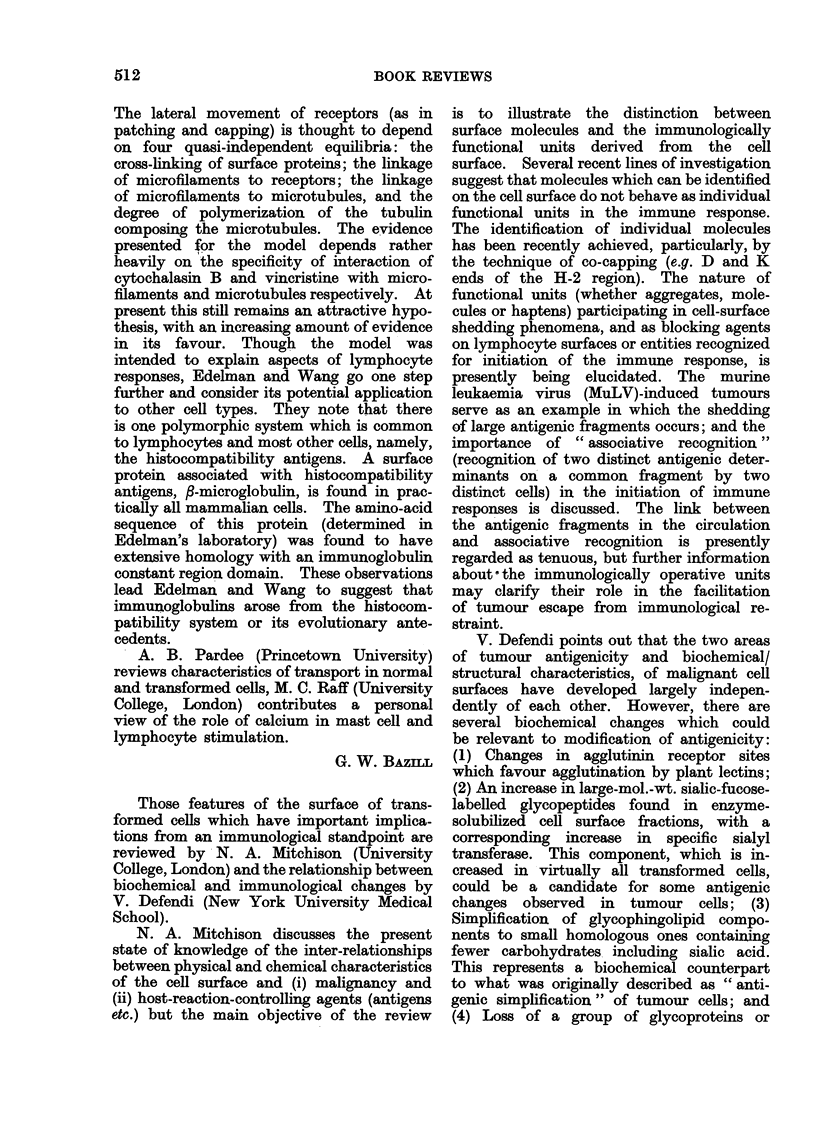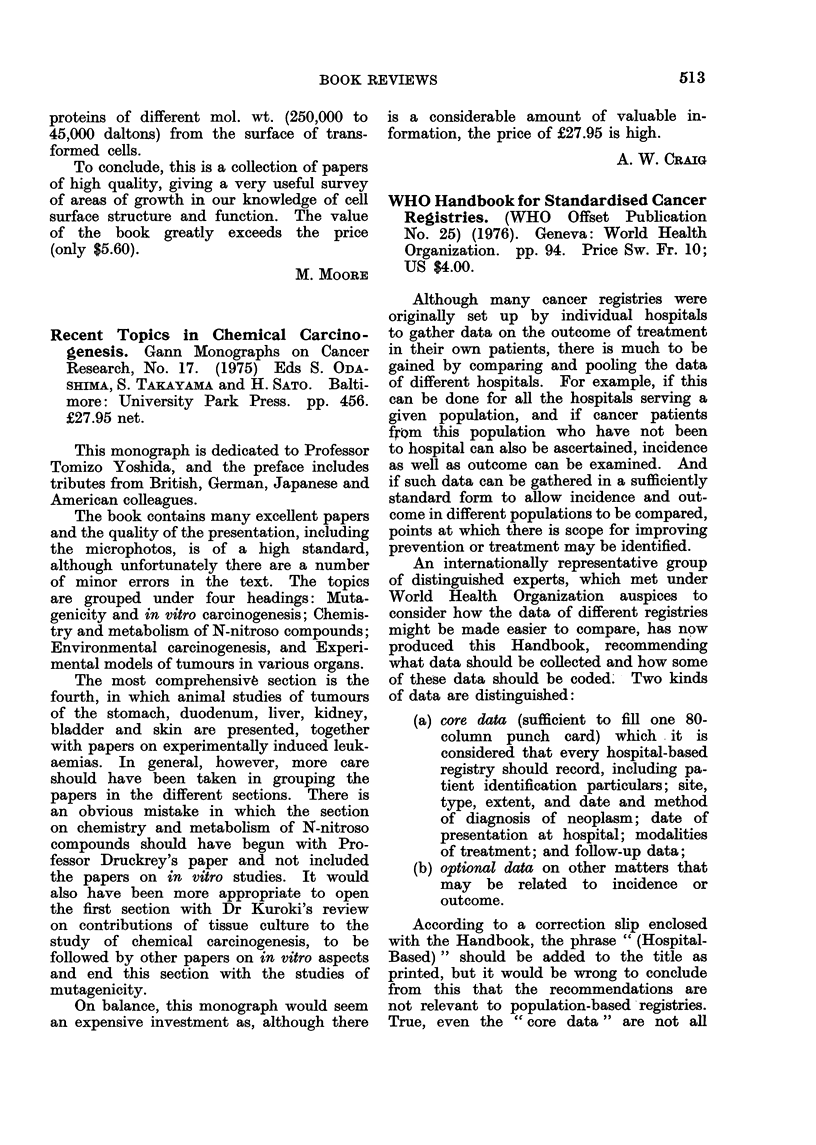# Cell Surfaces and Malignancy

**Published:** 1977-04

**Authors:** M. Moore


**Cell Surfaces and Malignancy** Ed. P. T. MORA, E. D. KORN, V. DEFENDI and P. W. ROBBINS (1976). Bethesda, Maryland: National Institutes of Health. 293 pp. Price $5.60 net.

This is a report of the proceedings of a workshop conference on cell surfaces and malignancy held at the National Institutes of Health in September 1974. As the title implies, the topics are diverse. The physical structure and chemistry of the plasma membrane, the role of the membrane in cellular interactions and the surface characteristics of malignant cells are about equally represented in the papers.

M. Neville (National Institutes of Health) opens the proceedings with a review of methods for preparing plasma membranes and criteria of purity. He emphasizes that different cell types may behave in totally different ways when subjected to the same membrane preparation procedure. Consequently, the interpretation of experiments in which, for example, plasma membranes of normal and transformed cells are compared, is far from straightforward. Anyone initiating such studies would be well advised to read this paper, as Dr Neville points out the many pitfalls and indicates how some, at least, may be avoided.

Electron spin resonance, nuclear magnetic resonance and fluorescence studies have provided a great deal of the physical basis for the fluid mosaic model of the plasma membrane. The use of physical probes and the information they have provided is reviewed with characteristic lucidity by J. C. Metcalfe (University of Cambridge). He also describes elegant studies on the influence of lipid association on the function of purified ATPase from thes arcoplasmic reticulum.

Evidence for the asymmetric distribution of phospholipids in the erythrocyte membrane is presented by R. F. A. Zwaal (University of Utrecht) and G. V. Marinetti (University of Rochester). Labelling of intact erythrocytes with the nonpermeable reagent formyl methionine methyl phosphate first led M. Bretscher to propose an asymmetry of phospholipids in the membrane. Similar results are reported by G. V. Marinetti using the probe, 2,3,5-trinitrobenzene sulphonate. He concludes that at least 70% (probably 90%) of phosphatidyl ethanolamine, and 100% of phosphatidyl serine are located on the inner surface of the erythrocyte membrane. In a complementary approach, R. F. A. Zwaal compared the effect of sphingomyelinase and phospholipase $A_2$ (which hydrolyses phosphatidyl choline) on sealed and unsealed erythrocyte membranes. The conclusions from these studies are in broad agreement with those from chemical probe experiments: phosphatidylcholine and sphingomyelin are external, while phosphatidyl-serine and phosphatidylethanolamine are internal. In the discussion, M. Bretscher gives a useful summary of the location of erythrocyte membrane proteins, and speculates that phospholipid asymmetry may be inherent in the mechanism of membrane biosynthesis.

In a paper intended to present models and ideas rather than primary data, G. M. Edelman and J. L. Wang propose a model for lymphocyte receptors which supposes that they are anchored, *via* actin microfilaments, to a cytoskeleton of microtubules.

The lateral movement of receptors (as in patching and capping) is thought to depend on four quasi-independent equilibria: the cross-linking of surface proteins; the linkage of microfilaments to receptors; the linkage of microfilaments to microtubules, and the degree of polymerization of the tubulin composing the microtubules. The evidence presented for the model depends rather heavily on the specificity of interaction of cytochalasin B and vincristine with microfilaments and microtubules respectively. At present this still remains an attractive hypothesis, with an increasing amount of evidence in its favour. Though the model was intended to explain aspects of lymphocyte responses, Edelman and Wang go one step further and consider its potential application to other cell types. They note that there is one polymorphic system which is common to lymphocytes and most other cells, namely, the histocompatibility antigens. A surface protein associated with histocompatibility antigens, $\beta$-microglobulin, is found in practically all mammalian cells. The amino-acid sequence of this protein (determined in Edelman's laboratory) was found to have extensive homology with an immunoglobulin constant region domain. These observations lead Edelman and Wang to suggest that immunoglobulins arose from the histocompatibility system or its evolutionary antecedents.

A. B. Pardee (Princetown University) reviews characteristics of transport in normal and transformed cells, M. C. Raff (University College, London) contributes a personal view of the role of calcium in mast cell and lymphocyte stimulation.

G. W. Bazill

Those features of the surface of transformed cells which have important implications from an immunological standpoint are reviewed by N. A. Mitchison (University College, London) and the relationship between biochemical and immunological changes by V. Defendi (New York University Medical School).

N. A. Mitchison discusses the present state of knowledge of the inter-relationships between physical and chemical characteristics of the cell surface and (i) malignancy and (ii) host-reaction-controlling agents (antigens *etc.*) but the main objective of the review is to illustrate the distinction between surface molecules and the immunologically functional units derived from the cell surface. Several recent lines of investigation suggest that molecules which can be identified on the cell surface do not behave as individual functional units in the immune response. The identification of individual molecules has been recently achieved, particularly, by the technique of co-capping (*e.g.* D and K ends of the H-2 region). The nature of functional units (whether aggregates, molecules or haptens) participating in cell-surface shedding phenomena, and as blocking agents on lymphocyte surfaces or entities recognized for initiation of the immune response, is presently being elucidated. The murine leukaemia virus (MuLV)-induced tumours serve as an example in which the shedding of large antigenic fragments occurs; and the importance of "associative recognition" (recognition of two distinct antigenic determinants on a common fragment by two distinct cells) in the initiation of immune responses is discussed. The link between the antigenic fragments in the circulation and associative recognition is presently regarded as tenuous, but further information about the immunologically operative units may clarify their role in the facilitation of tumour escape from immunological restraint.

V. Defendi points out that the two areas of tumour antigenicity and biochemical/structural characteristics, of malignant cell surfaces have developed largely independently of each other. However, there are several biochemical changes which could be relevant to modification of antigenicity: (1) Changes in agglutinin receptor sites which favour agglutination by plant lectins; (2) An increase in large-mol.-wt. sialic-fucose-labelled glycopeptides found in enzyme-solubilized cell surface fractions, with a corresponding increase in specific sialyl transferase. This component, which is increased in virtually all transformed cells, could be a candidate for some antigenic changes observed in tumour cells; (3) Simplification of glycophingolipid components to small homologous ones containing fewer carbohydrates including sialic acid. This represents a biochemical counterpart to what was originally described as "antigenic simplification" of tumour cells; and (4) Loss of a group of glycoproteins or proteins of different mol. wt. (250,000 to 45,000 daltons) from the surface of transformed cells.

To conclude, this is a collection of papers of high quality, giving a very useful survey of areas of growth in our knowledge of cell surface structure and function. The value of the book greatly exceeds the price (only $5.60).

M. MOORE